# Gene expression levels associated with impaired immune response and increased proliferation could serve as biomarkers for women following cervical cancer screening programmes

**DOI:** 10.3389/fimmu.2024.1507193

**Published:** 2024-12-06

**Authors:** Irene T. Ovestad, Ingvild Dalen, Kristiane Soreng, Saleha Akbari, Morten Lapin, Emiel AM Janssen, Marie Austdal, Ane Cecilie Munk, Einar Gudlaugsson

**Affiliations:** ^1^ Department of Pathology, Stavanger University Hospital, Stavanger, Norway; ^2^ Section of Biostatistics, Department of Research, Stavanger University Hospital, Stavanger, Norway; ^3^ National HPV Reference laboratory Department of Microbiology and Infection Control, Akershus University Hospital, Lørenskog, Norway; ^4^ Department of Haematology and Oncology, Stavanger University Hospital, Stavanger, Norway; ^5^ Department of Chemistry, Bioscience and Environmental Technology, University of Stavanger, Stavanger, Norway; ^6^ Department of Gynaecology, Sorlandet Hospital, Kristiansand, Norway

**Keywords:** HPV, NGS, qPCR, cervical precancer, biomarkers, immunology, ROC-AUC

## Abstract

Human papilloma virus (HPV) infections vary in their oncogenic potential, and whether an infection progresses to cervical intraepithelial neoplasia (CIN) also depends on the immune response. Therefore, the aim of the present study was to explore biomarkers related to the immune system and cell proliferation, in combination with HPV classified as having high (HOP) or low oncogenic potential (LOP), that can possibly guide a more accurate identification of women following cervical cancer screening programmes in need for immediate follow-up with a biopsy. A next-generation sequencing transcriptomic immune profile analysis applied to 28 persistent CIN3 lesions and 14 normal biopsies identified four genes, the immune markers *ARG1* and *HLA-DQB2* and the tumour markers *CDKN2A* and *KRT7*, as possible markers for differentiating between CIN3 and normal tissue. To validate these findings, analysis of the relative gene expression of these markers by use of reverse transcriptase real-time quantitative polymerase chain reaction was performed in an independent cohort of 264 (82 normal, 64 CIN1, and 118 CIN2/CIN3) biopsies, and the data were combined with information on the HOP- or LOP-HPV identified in the biopsies. Statistical analysis was performed with receiver operating characteristic curves, reporting area under the curve (AUC) with 95% confidence intervals (CIs), and logistic regression. Statistically significantly higher median expression levels of *CDKN2A* (*p* < 0.001) and *KRT7* (*p* = 0.045) and significantly lower expression of *ARG1* (*p* = 0.012) were found in biopsies with HOP-HPV infections, with no difference detected for *HLA-DQB2* (*p* = 0.82). Models using expression levels of *CDKN2A* (AUC, 0.91; 95% CI, 0.86–0.95), *KRT7* (0.86, 0.81–0.91), or *ARG1* (0.78, 0.70–0.85) together with HOP/LOP-HPV class were significantly better than HPV class alone (0.72, 0.66–0.79) in discriminating CIN2/3 versus CIN1 (*p* < 0.001, *p* < 0.001, and *p* = 0.014, respectively).

## 1 Introduction

Cervical intraepithelial neoplasia (CIN) is a premalignant condition caused by persistent human papilloma virus (HPV) infections and is classified as CIN1, CIN2, or CIN3 based on increased severity. Most sexually active women are exposed to an HPV infection, but less than 10% develop persistent infections associated with a higher risk of developing cervical cancer. Ninety per cent of the infections are cleared naturally within 1–2 years without any harm (1). This suggests possible individual variation regarding the oncogenic potential and reduced immune defences provoked by different HPV types. Many national screening programmes have implemented primary HPV testing as a substitute for primary cytology screening. The advantage is to detect women at risk in an early phase, but at the expense of low specificity, resulting in several unnecessary biopsies taken from HPV-positive women, diagnosed as CIN1 or normal and not subjected to immediate risk for progression to cancer (2). In addition to evoking needless anxiety among many women, this also implies unnecessary use of staff resources in pathology departments. Many HPV assays used for routine screening specifically detect HPV16 and 18, while a group of 12 other high-risk HPVs (hrHPVs) is often detected as groups with highly variable oncogenic potential (3). The use of extended genotyping analysis to detect genotypes with the most oncogenic potential in the group “12 others” is being introduced in Scandinavian countries (4).

In an evaluation by the International Agency for Research on Cancer (IARC) (5), 12 different HPV types are defined as hrHPV (HPV16, 18, 31, 33, 35, 39, 45, 51, 52, 56, 58, and 59), while a group of 13 HPV types are characterized as possible/probably high-risk (phrHPV) (HPV26, 30, 34, 53, 66, 67, 68, 69, 70, 73 82, 85, and 97). However, the oncogenic potential still shows large variation within these categories. HPV6 and 11 are characterized as low-risk (lrHPV) and non-carcinogenic.

Prior to the prophylactic HPV vaccination era, a population-based study in four Nordic countries evaluated the distribution of HPV genotypes from women with CIN2, CIN3, or cervical cancer. The most prevalent HPV types detected in CIN2 and CIN3 were HPV16 (35.9% and 50.2%, respectively) and HPV31 (10.9% and 12.1%, respectively). HPV33, 52, and 18 were overall less prevalent, with 8.4%, 13.2%, and 7.8% in CIN2 and 12.3%, 8.7%, and 8.6% in CIN3, respectively. However, the HPV types that were most prevalent in cancer were HPV16 (48.8%) and HPV18 (15.3%) (6).

In the first screening round in a long-term follow-up HPV primary screening trial in Sweden, the hrHPV types HPV16, 18, 31, 33, 45, 52, and 58 were found to contribute 80.8% and 88.2% for CIN2+ and CIN3+ development, respectively, while less than 10% cumulative incidence risk for CIN2+ was found for hrHPV types HPV35, 39, 51, 56, and 59 and phrHPV types HPV66 and 68 (7). Furthermore, a worldwide, retrospective, cross-sectional study detected single phrHPV types HPV26, 53, 66, 67, 68, 70, 73, and 82 in approximately 3% of the tested cervical cancer cases (8). The two most common lrHPV types, HPV6 and 11, have been found to be mostly involved in laryngeal squamous cell carcinoma and only sporadically in cervical cancer (9). The carcinogenicity of different HPV types may also depend on the viral load. A study of HPV copy number, as measured by quantitative polymerase chain reaction (PCR)-based fluorescent assays, found differences in copy number between various HPV types (HPV16, 18, 31, and 45) and a significant increase in accordance with more severe disease as detected in normal, CIN1, and CIN2/3 biopsies (10).

Even though an infection with HPV is the underlying cause, it is not always sufficient for developing cervical cancer (11, 12). In order to successfully reproduce viral copies, hrHPV types have developed the ability to downregulate tumour suppressors and thereby interfere with the host cell cycle and immune responses.

During cervical cancer progression, the HPV oncogene E7 entails the overexpression of the cyclin-dependent kinase inhibitor p16INK4a, encoded by *CDKN2A*. The result is inactivation and degradation of the cell cycle regulatory retinoblastoma protein (pRb). Furthermore, the E6 oncogene mediates degradation of the tumour suppressor p53 with subsequent attenuated cell cycle arrest in response to DNA damage. Immediately after infection, an upregulation of host cell cycle and proliferation-related genes has been observed (13, 14).

Additionally, hrHPV oncogenes E6 and E7 have developed the ability to epigenetically regulate host gene expression by interfering with the activity of the DNA methyl transferase DNMT1, a key enzyme responsible for maintaining the methylation pattern of promoter CpG islands (15). Upregulated methylation patterns of the type III interferon-γ (*IFN-γ*) promoter and subsequently lower levels of IFN-γ were found in cervical cancer, as compared to normal, CIN1, CIN2, and CIN3 tissues (16, 17). The result is diminished favourable immune responses with downregulated development of effector T cells and adapted immunity with antigen-specific memory T cells (18).

Type I *IFN-κ* is constitutively expressed in uninfected cervical keratinocytes but was found to be downregulated by E6 in HPV16- and 18-positive cervical carcinoma cells due to increased methylation of the promoter. Diminished IFN-κ could be an early event during cervical carcinogenesis, affecting the expression of the tumour suppressor p53, IFN regulatory factors (IRFs), and MxA (19). IRF acts as a link between cellular responses and oncogenesis (20), and a downregulation would affect the development and function of several types of immune cells. Downregulation of the antiviral activity for the interferon-induced MxA GTPase would also have an impact on the immune response (21). Moreover, *CXCL14* is a chemokine with downregulated expression due to promoter hypermethylation by hrHPV E7. Reduced expression of *CXCL14* in HPV-infected cells affects the activation and migration of dendritic cells (DCs), causing immune evasion and suppressed antitumour immune responses with lower numbers of natural killer (NK) cells, CD4+ helper T cells, and cytotoxic CD8+ T cells (22, 23).

In summary, when expressed simultaneously, E6 and E7 can downregulate immune responses, immortalize human cells by blocking their exit from the cell cycle, and prevent cell cycle arrest of cells harbouring damaged DNA. The result is enhanced proliferation of infected cells with hundreds of HPV copies per cell in a carcinogenic environment with a risk of developing cancer.

The objective of the present study was to explore biomarkers to distinguish between women following a cervical cancer screening programme (CCSP) in need of immediate follow-up with biopsy and those who would benefit from a less invasive follow-up with cytology. We tested the use of expression levels for *CDKN2A*, *KRT7*, *ARG1*, and *HLA-DQB2*, identified through next-generation sequencing (NGS) analysis, for their ability to discriminate CIN2/CIN3 versus CIN1/and or normal biopsies, both alone and in combination with the distinct oncogenic potential of the HPV types.

## 2 Materials and methods

### 2.1 Biological material

Biological material collected at Stavanger University Hospital, Norway (SUH), from three different cohorts was included in the study. The women were referred to the gynaecologic outpatient clinic for histologic follow-up of an abnormal or HPV-positive cytology test. In cohort 1, 254 women were prospectively recruited at the gynaecologic outpatient clinic between January 2007 and December 2008; in cohort 2, 354 women were similarly recruited between March 2015 and June 2018. Cohorts 1 and 2 have been thoroughly described in earlier studies (24, 25). Cohort 3 consists of 111 biopsies collected retrospectively at the Pathology Department (SUH) between November 2017 and June 2018. The women were identified to have an abnormal and/or HPV-positive cytology specimen and were referred for a biopsy as recommended by the CCSP.

**Figure 1:**
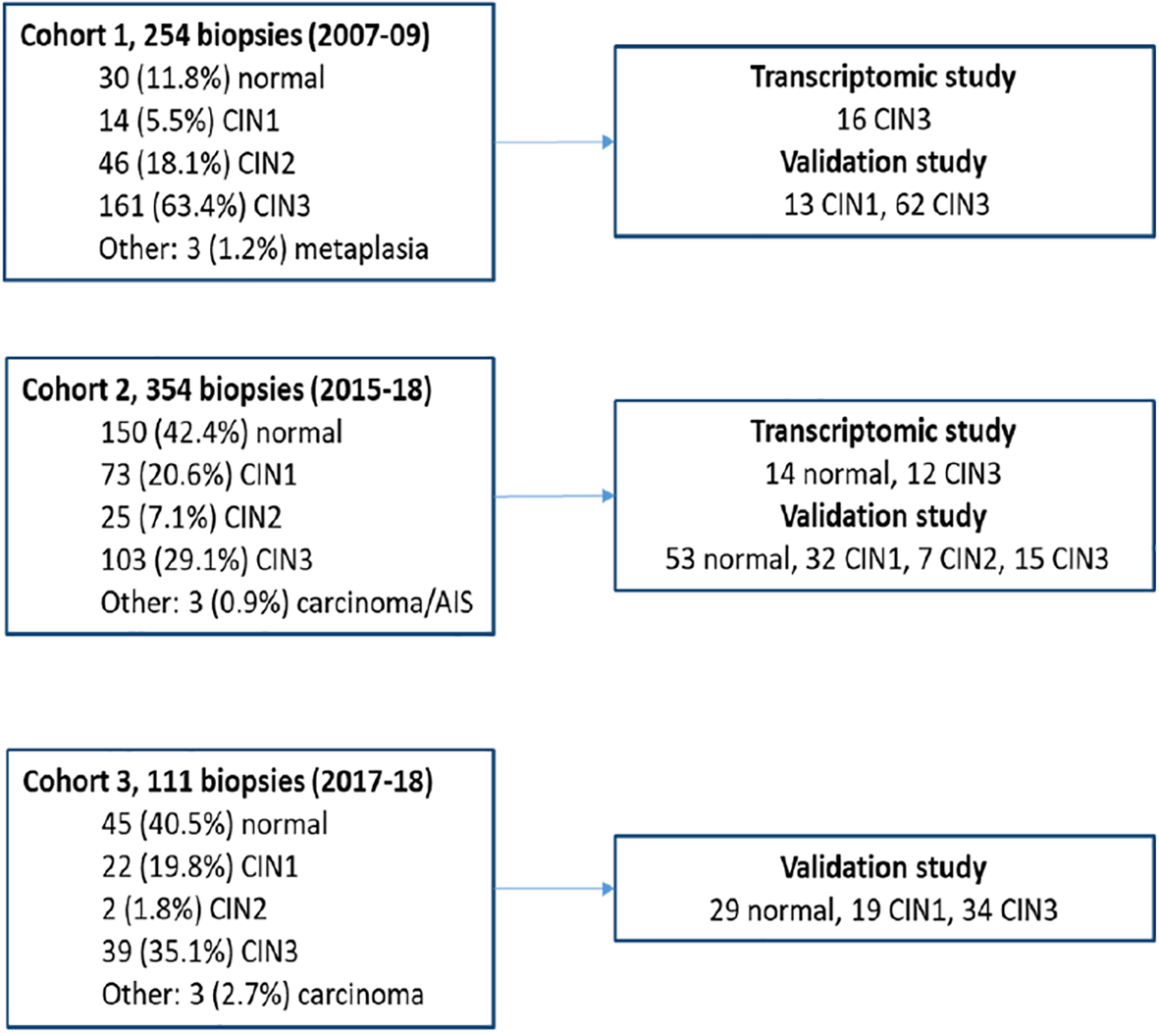
Overview of the study cohorts and biopsies used in the transcriptomic and validation studies.

An expert pathologist evaluated all haematoxylin and eosin (H&E)-stained slides from the formalin-fixed paraffin-embedded (FFPE) biopsies, supported by immunohistochemical (IHC) staining of Ki-67 and p16. Interpretation by the pathologist regarding the lesion and sufficient high-quality RNA as measured by the RNA quantification assay supported the selection of 42 biopsies used in the transcriptomic study (14 normal and 28 CIN3) and 264 biopsies for use in the validation study (82 normal, 64 CIN1, 7 CIN2, and 111 CIN3). **Figure 1** gives an overview of the study cohorts. Written informed consent was received from all patients by inclusion. The studies were approved by the Norwegian Regional Ethics Committee (REC West) (2016/805, 2019/264, 2012/1292, 2019/10399, and 2022/10399).

### 2.2 Cytology diagnoses

As described earlier and in accordance with the former national guidelines of the Norwegian cervical cancer screening programme (25), the baseline cytology specimens were categorized as negative for intraepithelial lesion malignancy (NILM), atypical squamous cells of undetermined significance (ASC-US), atypical glandular cells of undetermined significance (AGUS), low-grade squamous intraepithelial lesion (LSIL), atypical squamous cells, cannot rule out high-grade squamous intraepithelial lesion (ASC-H), or high-grade squamous intraepithelial lesion (HSIL). Regardless of diagnosis, in total, 69.8% were proven HPV positive, whereas 26.9% were diagnosed as HSIL/ASC-H and in line with the Norwegian national guidelines were not HPV tested. Additionally, three cases diagnosed as AS-CUS, five as AGUS, and one normal, in total 3.4%, were not HPV tested.

### 2.3 Isolation of RNA and DNA and transcription of cDNA

RNA and DNA were isolated from FFPE biopsies. For CIN1, CIN2, and CIN3 biopsies, nucleic acids were isolated from (5–10) × (4–5 µm) sections from the FFPE tissue block. The sections were macro-dissected from the most severe dysplastic area of the epithelium and the adjacent stroma, as marked by an expert pathologist. For normal biopsies, nucleic acids were isolated from (2–5) × (4–5 µm) sections comprising all the biopsies in the FFPE tissue block. To assure quality for the CIN1 and CIN2/CIN3 diagnoses, adjacent sections to the sections used for isolation were H&E-stained and examined by the pathologist.

For cohort 1, the miRNeasy FFPE kit (Qiagen, Hilden, Germany) was used for RNA isolation and the E.Z.N.A^®^ Tissue DNA Kit (Omega Bio-tek Inc., Norcross, GA, USA) for DNA isolation. For cohorts 2 and 3, the “Recover all total nucleic acid isolation” kit (Thermo Fisher Scientific, Waltham, MA, USA) was used for simultaneous isolation of RNA and DNA. An RNA quantification assay (Thermo Fisher Scientific, Pub. No. MAN0015867) was applied to ensure the exact amplifiable RNA concentrations from each of the FFPE samples. The assay is a one-step reverse transcriptase real-time quantitative PCR (RT-qPCR) procedure performed on the LightCycler^®^ 480 System (Roche Diagnostics, Rotkreuz, Switzerland) and has been thoroughly explained in former publications (24, 25). The Superscript Vilo cDNA synthesis kit (Thermo Fisher Scientific) was used for the transcription of 10 ng total RNA, as calculated by functional RNA quantification. For all methods, the procedure was performed according to the protocol of the manufacturer. To ensure no genomic DNA contamination of the RNA samples, a non-reverse transcriptase (noRT) control was included in all batches of samples for cDNA transcription.

### 2.4 HPV genotyping and categorization

From cohort 2, 34 of 150 normal biopsies were genotyped using the INNO-LiPA HPV detection system (Fujirebio Europe N.V., Gent, Belgium). INNO-LiPA is an automated line probe assay for the detection of 32 different genotypes including high-risk, low-risk, and probably/possible high-risk HPV (26). The analysis was performed according to the manufacturer’s recommendations.

Anyplex II HPV28 (Seegene, Seoul, South Korea) was performed on DNA isolated from all CIN1, CIN2, and CIN3 biopsies as recommended by the manufacturer (Seegene, South Korea). The assay simultaneously detects 28 HPV genotypes using HPV-specific dual priming oligonucleotides (DPOs) (27) in a multiplex real-time PCR, coupled with the TOCE™ technology (28). HPV genotyping with Anyplex II HPV28 was performed on a CFX96 real-time PCR instrument (Bio-Rad, Hercules, CA, USA; provided together with the Anyplex II HPV28 kit). Data analysis was carried out using the Seegene viewer software according to the manufacturer’s instructions.

Based on results from the IARC working group (5), the International Human Papillomavirus Reference Center (29), and previous longitudinal studies mentioned above (6-8), the observed HPV types were grouped according to their oncogenic potential either as high oncogenic potential HPV (HOP-HPV) (HPV16, 18, 31, 33, 45, 52, and 58) or as low oncogenic potential HPV (LOP-HPV) (HPV26, 35, 39, 51, 53, 56, 59, 66, 68, 69, 73, and 82). The LOP-HPV group also includes non-cancerous lrHPV (HPV6, 42, 61, and 89) and HPV negativity, presumably with low HPV copy number and not detectable by HPV genotyping assays used in routine screening of women attending CCSP (3, 4). If more than one HPV type was detected and at least one of them was in the HOP-HPV group, the sample was categorized as HOP-HPV.

### 2.5 Next-generation sequencing analysis

The Oncomine™ Immune Response Research Assay (Thermo Fisher Scientific) targets and quantifies the expression levels of a panel of 398 immune response-related genes, including 10 housekeeping genes (30). According to the Ion Chef system protocol (Thermo Fisher Scientific), automated library preparation of the samples, each containing 10 ng/µL cDNA, was performed in batches of eight libraries of eight samples. The library concentrations were measured by use of the Ion Library *TaqMan* quantitation kit, and the Ion OneTouch™ 2 System (Thermo Fisher Scientific) was used to prepare the enriched, template-positive Ion PI™ Ion Sphere™ Particles (ISPs).

In total, 10 µL, diluted 1/6 with Tris Low EDTA (Low TE) buffer, from each library were combined and further diluted to 100 pM. The combined libraries were used as a template in the emulsion PCR on the Ion OneTouch™ 2 Instrument using the Ion PI™ Hi-Q™ OT2 200 kit (Thermo Fisher Scientific). Target sequencing was performed on an Ion Proton instrument using the Ion PI™ Hi-Q™ Sequencing 200 chemistry and an Ion PI™ chip (Thermo Fisher Scientific). The procedure has been thoroughly explained in former publications (24, 25).

### 2.6 Transcriptomic analysis

Subsequent to the target sequencing, the results were downloaded to the Affymetrix Transcriptome Analysis Console (TAC) (Thermo Fisher Scientific) for further data analysis of the 42 samples used in the transcriptomic study. Mean housekeeping gene scaled log2 count data from the 398 genes in the Oncomine Immune Response panel were obtained from the Torrent Suite™ Software (Thermo Fisher Scientific). Differential expressions between the two experimental groups, 28 CIN3 and 14 normal biopsies, were analysed by use of the TAC version 4.0.2. An exploratory grouping analysis provides gene-level analysis from small starting material, and the workflow involves a two-step process. The first step generates clusters from the data followed by expression analysis to generate fold-changes, *p*-values, and false discovery rate (FDR)-adjusted *p*-values. The analysis strategy has been thoroughly explained in earlier publications (24, 25).

### 2.7 Real-time quantitative PCR

Real-time quantitative PCR (qPCR) was performed on Quant Studio 6 (Applied Biosystems, Thermo Fisher Scientific, Waltham, MA, USA). The qPCR was performed using TaqMan Fast Advanced Master Mix (Life Technologies, Carlsbad, CA, USA) and TaqMan assays targeting CDKN2A (Hs00923894), ARG1 (Hs00163660), KRT7 (Hs00559840), and HLA-DQB2 (Hs00745107). Based on the results from the NGS analysis, the housekeeping gene ABCF1 (hs01073518) was chosen as the internal reference. The normalized Cq method (2^−ΔCq^) was used to calculate the relative gene expression of each gene as measured in RNA isolated from normal, CIN1, CIN2, and CIN3 biopsies (31). To ensure that RNA was not contaminated with genomic DNA, noRT controls were included in all batches for cDNA synthesis and analysed by use of RT-PCR using the TaqMan genotyping MasterMix (Thermo Fisher Scientific) and the TaqMan Copy Number Assay for the housekeeping gene ABCF1. All analyses were conducted according to the manufacturer’s instructions.

### 2.8 Statistical analysis

Clustering analysis was performed with expression levels of the four biomarkers and illustrated in a heatmap including diagnosis (CIN2/3 vs. normal/CIN1) and the HPV group (HOP-HPV vs. LOP-HPV). Expression values of zero were imputed as half the minimum observed value. Following log transformation, expression values were scaled by subtracting the row (gene) mean expression and dividing by the standard deviation. Patients were clustered using complete clustering with Euclidian distance measures.

The ability to discriminate between CIN2/3 vs. normal and CIN1 was assessed using receiver operating characteristic (ROC) curves. For any one biomarker, the observed expression values went into the ROC analysis, from which we report the area under the ROC curve (AUC) and 95% confidence interval (CI). In situations where lower expression levels predicted higher risks of CIN2/3, we reversed the outcome (in essence detecting e.g., CIN1 vs. CIN2/3) and reported the corresponding AUC > 0.5. For models combining different biomarkers, binary logistic regression was used with CIN2/3 (yes/no) as outcome and log-transformed expression levels as predictors. Predicted probabilities (of CIN2/3) were estimated from the fitted model and went into the ROC analysis. Log transformation was conducted to stabilize the models by reducing the influence of outliers.

Notice that for the univariable ROC analyses, the log transformation of the predictor has no impact on the estimates of AUC. A comparison of different models for the same outcome was performed using the DeLong test, which allows for the fact that compared AUCs are assessed in the same data. A comparison of median expression levels between groups of participants was performed with quantile regression.

The clustered heatmap was generated using the pheatmap package v. 1.0.12 in R v. 4.2.3. ROC and regression analyses were performed in Stata v. 17, with functions roctab, logit, roccomp, allpossible, and qreg.

## 3 Results

### 3.1 Patient characteristics

In total 42 biopsies (16 CIN3 from cohort 1, and 12 CIN3 and 14 normal biopsies from cohort 2) were used for the NGS transcriptomic analysis. According to the former guidelines of the Norwegian national screening programme, the women, with a mean age of 33 years (range 26–51), were referred for follow-up with punch biopsy after an abnormal and/or HPV-positive cytology, with a median of 42 days [interquartile range (IQR), 37–58] prior to the biopsies. The median total follow-up of this cohort was 1,423 days (IQR, 1,291–2,297).

The mean age in the combined validation study cohort comprising 264 biopsies (82 normal, 64 CIN1, 7 CIN2, and 111 CIN3) was 35 years (range 23–71). A registered abnormal and/or HPV-positive cytology, defined as baseline cytology, was taken at a median of 41 days (IQR, 34–55) prior to the biopsy. The median total follow-up of the study cohort was 1,149 days (IQR, 694–1,677), counted from the biopsy collection until the last follow-up in February 2012 for cohort 1 and May 2023 for cohorts 2 and 3.

### 3.2 HPV genotypes

In the transcriptomic study, five HPV types with low oncogenic potential (LOP-HPV) and nine HPV types with high oncogenic potential (HOP-HPV) were detected in the normal biopsy group, while one LOP-HPV and 27 HOP-HPV were found in the CIN3 group.

In the validation cohort, HOP-HPV were detected in 127 (70%) of the 182 CIN-positive samples, and the distribution of HOP- and LOP-HPV was similar between the cohorts. In a cohort of 34 HPV-tested normal biopsies, 21 (62%) were HOP-HPV positive, 6 (18%) were LOP-HPV positive, and 7 (21%) were HPV negative. Thirteen of these were included in the current study: 10 (77%) HOP-HPV and 3 (23%) LOP-HPV. **Table 1** gives an overview of the different biopsy diagnoses with the corresponding HPV group detected in the biopsy and the different cytology findings. Overall, the majority (101 of 118; 86%) of the CIN2/CIN3 biopsies were infected with HOP-HPV and, to a large extent, independently of the cytology findings prior to the biopsy. In the CIN1 group, the distribution was more even, with 26 of 64 (41%) HOP-HPV overall and much the same in all cytology groups apart from for ASC-H/HSIL with two of nine (22%) HOP-HPV.

**Table 1:** Cytology results in baseline cytology and HPV genotypes and diagnoses in follow-up biopsies included in the validation study.

Baseline cytology and HPV genotypes detected in biopsy	Biopsy diagnosis			
	Normal^a^	CIN1	CIN2	CIN3
**Total**	**N = 13**	**N = 64**	**N = 7**	**N = 111**
HOP-HPV	10 (77%)	26 (41%)	7 (100%)	94 (85%)
LOP-HPV	3 (23%)	38 (59%)	–	17 (15%)
**NILM** ^b^	**N = 7**	**N = 21**	**N = 2**	**N = 6**
HOP-HPV	5 (71%)	10 (48%)	2 (100%)	5 (83%)
LOP-HPV	2 (29%)	11 (52%)	–	1 (17%)
ASC-US/AGUS^b^	**N = 2**	**N = 17**	–	**N = 13**
HOP-HPV	1 (50%)	6 (35%)	–	11 (85%)
LOP-HPV	1 (50%)	11 (65%)	–	2 (15%)
**LSIL**	**N = 2**	**N = 17**	**N = 1**	**N = 15**
HOP-HPV	2 (100%)	8 (47%)	1 (100%)	13 (87%)
LOP-HPV	–	9 (53%)	–	2 (13%)
**ASC-H/HSIL**	**N = 2**	**N = 9**	**N = 4**	**N = 77**
HOP-HPV	2 (100%)	2 (22%)	4 (100%)	65 (84%)
LOP-HPV	–	7 (78%)	–	12 (16%)

a Only a subset of 13 normal biopsies were HPV genotyped.

b bNILM/ASC-US/AGUS were biopsied if they were HPV positive.

N is the number of cases in the different CIN and cytology categories; HPV, Human Papilloma Virus; HOP-HPV, High Oncogenic Potential HPV (HPV16, 18, 31, 33, 45, 52, 58); LOP-HPV, Low Oncogenic Potential HPV (HPV26, 35, 39, 51, 53, 56, 59, 66, 68, 73, and 82, including non-cancerous HPV, i.e., HPV6, 42, 53, 61, 69, and 89, and HPV negative); NILM, Negative for Intraepithelial Lesion Malignancy; ASC-US, Atypical Squamous Cells of Undetermined Significance; AGUS, Atypical Glandular cells of Undetermined Significance; LSIL, Low-grade Squamous Intraepithelial Lesion; ASC-H, Atypical Squamous Cells, Cannot Rule Out High Grade Squamous Intra-Epithelial Lesion; HSIL, High-grade Squamous Intraepithelial Lesion; CIN1-3, Cervical Intraepithelial Neoplasia grade 1-3.

### 3.3 NGS transcriptomic analysis

The gene expression of 398 genes demonstrated 22 differentially expressed genes between the normal and CIN3 groups with absolute values of fold-changes >2, *p*-values <0.05, and FDR-adjusted *p*-values <0.05. Thirteen genes were upregulated and nine were downregulated in CIN3 versus normal biopsies (**Table 2**). In CIN3 versus normal biopsies, the tumour markers *CDKN2A* and *KRT7* had the highest fold-change, while genes related to a favourable immune response, *ARG1* and *NCAM1*, had the lowest. Predominantly, genes related to a favourable immune response (*CX3CR1*, *HLA-DQA2*, *HLA-G*, *HLA-DQB2*, *HLA-F-AS1*, *CXCR3*, and *IL18*) were downregulated in CIN3 versus normal, whereas genes related to attenuated immune response were upregulated (*CCL20*, *CDK1*, *CXCL1*, *CXCL13*, *CCNB2*, *TNFRSF18*, and *POU2AF1*). Two genes related to a favourable immune response, *ARG1* and *HLA-DQB2*, and the tumour markers *CDKN2A* and *KRT7* were selected for validation with qPCR and further analysis.

**Table 2:** Twenty-two differentially expressed genes identified by the Oncomine™ Immune Response Research Assay as upregulated (13 genes) and downregulated (nine genes) in CIN3 biopsies vs. normal biopsies, with absolute values of fold-changes >2, p-values <0.05, and false discovery rate (FDR)-adjusted p-values <0.05.

*Gene symbol*	*Fold-change*	*p-Value*	*FDR p-value*	*Gene function*
*ARG1*	−34.39	6.19E−07	4.11E−05	Macrophage marker
*NCAM1*	−8.9	1.05E−05	0.0003	NK cell
*CX3CR1*	−4.45	0.0033	0.0323	Lymphocyte Infiltrate
*HLA-DQA2*	−4.44	0.0044	0.0383	Antigen processing
*HLA-G*	−3.68	0.0008	0.0097	Antigen processing
*HLA-DQB2*	−3.61	0.0004	0.0053	Antigen processing
*HLA-F-AS1*	−3.46	0.0038	0.0349	HLA-F antisense RNA1
*CXCR3*	−2.39	0.0031	0.0311	Proliferation
*IL18*	−2.02	0.0062	0.0471	T-cell regulation
*CDKN2A*	23.48	4.75E−14	1.89E−11	Tumour marker
*KRT7*	4.97	3.86E−09	7.68E−07	Tumour marker
*CCL20*	3.91	0.0004	0.0053	Chemokine (C-C motif) ligand 20
*CDK1*	3.69	3.79E−06	0.0001	Proliferation
*CXCL1*	3.63	0.0004	0.0055	Proliferation
*CXCL13*	3.43	0.0066	0.0489	B-cell regulation
*MELK*	3.41	1.53E−07	1.22E−05	Proliferation
*MKI67*	3.25	8.49E−06	0.0002	Proliferation
*TOP2A*	2.77	2.00E−06	8.84E−05	Epithelial–mesenchymal transition
*CCNB2*	2.74	4.75E−06	0.0001	Proliferation
*FOXM1*	2.48	0.0003	0.0053	Proliferation
*TNFRSF18*	2.46	6.55E−05	0.0014	Lymphocyte infiltrate (Tregs)
*POU2AF1*	2.43	0.0006	0.0081	B-cell marker

N = 42 (28 CIN3 and 14 normal).

Tregs, T-regulatory lymphocytes.

### 3.4 Validation

The relative gene expressions of *ARG1*, *HLA-DQB2*, *CDKN2A*, and *KRT7* as assessed in qPCR were tested for their potential to discriminate CIN2/CIN3 versus CIN1 and/or normal.

#### 3.4.1 Hierarchical clustering

**Figure 2:**
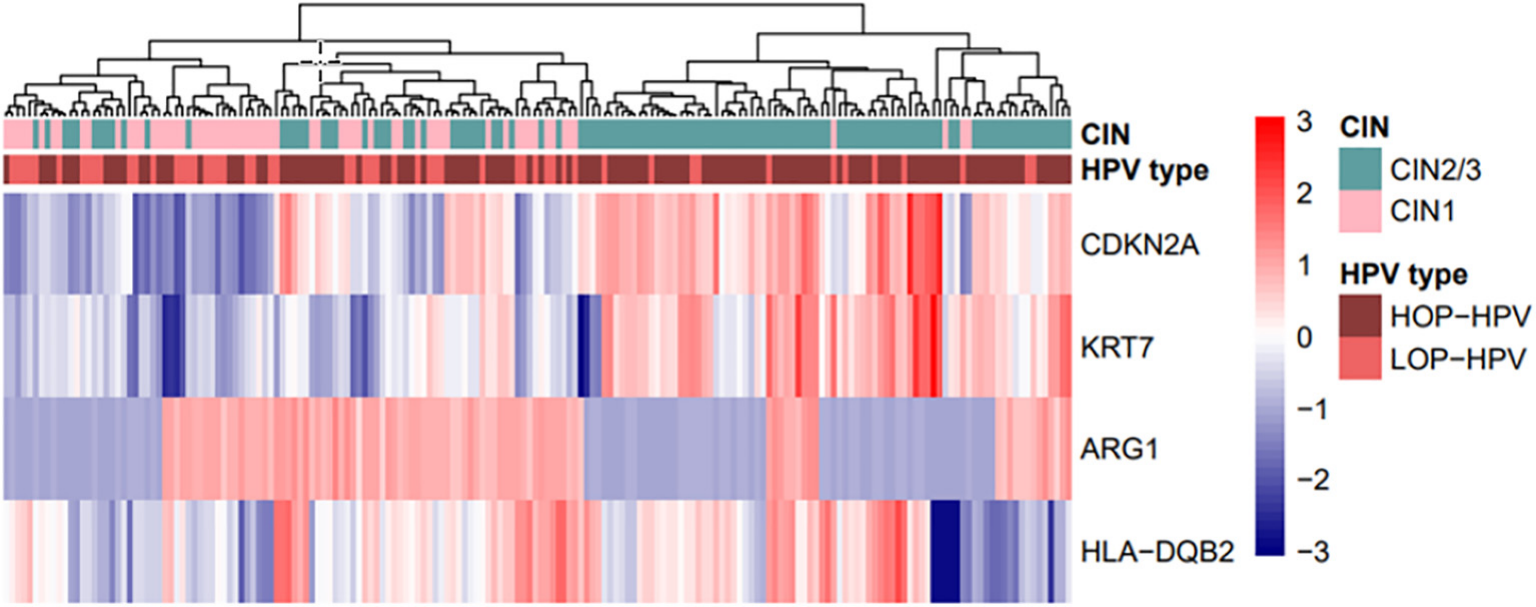
Hierarchical clustering based on the expression of four genes—*CDKN2A*, *KRT7*, *ARG1*, and *HLA-DQB2*—in biopsy samples, with correlation clustering distance and average linkage. The biopsy diagnosis, CIN2/3 (n = 118) vs. CIN1 (n = 64), and the HPV type, high oncogenic potential = HOP-HPV (n = 127) vs. low oncogenic potential = LOP-HPV (n = 55), are included in the plot. HPV, human papilloma virus.

Hierarchical clustering based on the expression of the four genes differentiated CIN1 from CIN2/3. Despite discrepancies, the majority of CIN1 have clusters with low expression of the tumour markers *CDKN2A* and *KRT7* and higher expression of *ARG1*. To a certain extent, the opposite is seen in CIN2/3 clusters with high expression of tumour markers and low expression of *ARG1*. Except for one clear cluster with low expression of *HLA-DQB2* in the CIN2/3 group, the expression of *HLA-DQB2* does not clearly differentiate between CIN1 and CIN2/3 (see **Figure 2**).

#### 3.4.2 ROC analyses with biomarker expression levels

For the detection of CIN2/CIN3 (n = 118) versus CIN1/normal (n = 146), the AUC values obtained were 0.93 for *CDKN2A* (95% CI, 0.90–0.96), 0.85 for *KRT7* (0.80–0.90), 0.68 for *ARG1* (reversed) (0.61–0.75), and 0.57 for *HLA-DQB2* (reversed) (0.50–0.64). The results demonstrated *CDKN2A* as a very good, *KRT7* as a good, and *ARG1* as a fairly good classifier for CIN3 versus normal or CIN1, while *HLA-DQB2* was not fitted for discriminating between the two groups as a stand-alone test.

**Figure 3:**
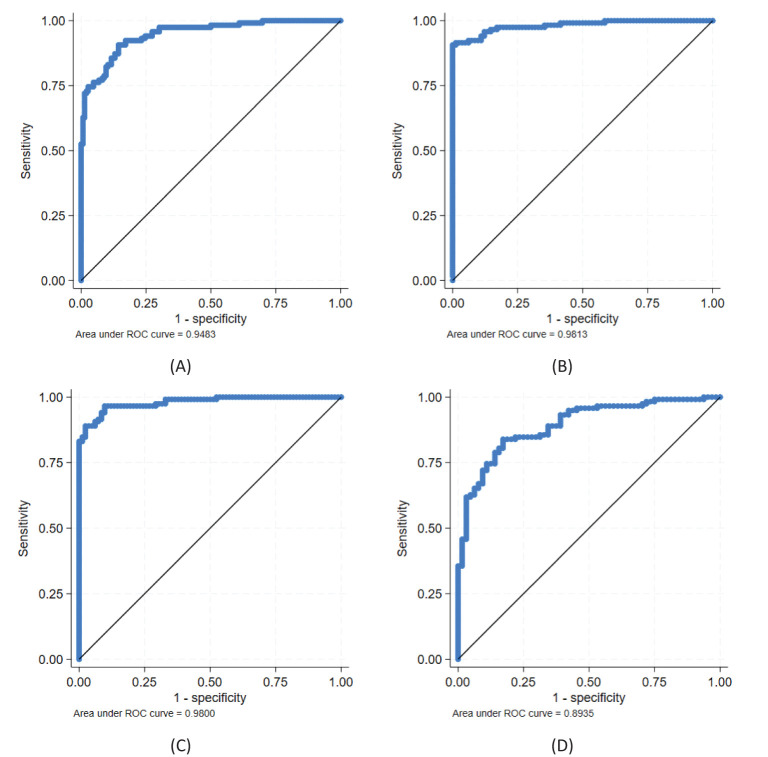
Ability of biomarkers to discriminate between CIN2/3 and CIN1 and/or normal; best models obtained with candidate genes *CDKN2A*, *KRT7*, *ARG1*, and *HLA-DQB2*: **(A)** *CDKN2A* + *ARG1* for discriminating CIN2/3 (n = 118) vs. CIN1/normal (n = 146), **(B)** *CDKN2A* + *ARG1* and **(C)** *CDKN2A* + *HLA-DQB2* for discriminating CIN2/3 vs. normal (n = 82), and **(D)** *CDKN2A* for discriminating CIN2/3 vs. CIN1 (n = 64). See further details in **Table 3**.

A combined model for all four biomarkers, i.e., (log-transformed) *CDKN2A*, *KRT7*, *ARG1*, and *HLA-DQB2* with AUC 0.95 (95% CI 0.93–0.97), showed statistically significantly better discrimination than *CDKN2A* alone (*p* = 0.027). However, the improvement came with the addition of *ARG1*, giving an AUC of 0.95 (0.92–0.97; *p* = 0.030 compared with only *CDKN2A*); any further improvement was deemed not statistically significant. The best model is illustrated in **Figure 3A**.

For the detection of CIN2/CIN3 (n = 118) versus normal (n = 82), the AUC for *CDKN2A* was 0.96 (95% CI, 0.94–0.99), *KRT7* 0.89 (0.85–0.94), *ARG1* 0.72 (0.65–0.80), and *HLA-DQB2* 0.69 (0.61–0.76). A combined model for all four biomarkers (log-transformed) with AUC 0.99 (0.97–1) was statistically significantly better for classification compared to *CDKN2A* alone (*p* = 0.027).

A model using *CDKN2A* and *ARG1* (AUC 0.98, 0.97–1; **Figure 3B**) or *CDKN2A* and *HLA-DQB2* (AUC 0.98, 0.97–1; **Figure 3C**) were both statistically significantly better than *CDKN2A* alone (*p* = 0.030 and *p* = 0.041, respectively), and no statistically significant differences were found between the two combinations (*p* = 0.76). Using all three biomarkers was also statistically significantly better than *CDKN2A* alone (AUC 0.98, 0.97–1; *p* = 0.036), but not statistically significantly better than a combination of either *CDKN2A* and *ARG1* or *CDKN2A* and *HLA-DQB2* (*p* = 0.37 and *p* = 0.24, respectively). Neither was the model using all four biomarkers statistically significantly better than these two combinations (*p* = 0.21 and *p* = 0.14, respectively).

For the classification of CIN2/CIN3 (n = 118) versus CIN1 (n = 64), the AUC for *CDKN2A* was 0.89 (95% CI, 0.85–0.94; **Figure 3D**), *KRT7* 0.80 (0.74–0.87), *ARG1* 0.62 (0.54–0.71), and *HLA-DQB2* 0.54 (0.46–0.63). The AUC for the model with all four biomarkers was 0.91 (0.87–0.95) and not statistically significantly better as compared to *CDKN2A* alone (*p* = 0.11). Neither was the best combination of two biomarkers, i.e., *CDKN2A* and *ARG1* with AUC 0.91 (0.86–0.95), statistically significantly better than *CDKN2A* alone (*p* = 0.17).

All results from the ROC analyses are summarized in **Table 3**.

**Table 3:** Discriminative ability of the expression of *CDKN2A*, *KRT7*, *ARG1*, and *HLA-DQB2* from qPCR analyses with regard to detecting CIN2/3 vs. CIN1 and/or normal and in combination with high/low oncogenic potential (HOP/LOP) HPV detected in the biopsies.

	CIN2/3 (n = 118) vs. CIN1/normal (n = 146)	CIN2/3 (n = 118) vs. normal (n = 82)	CIN2/3 (n = 118) vs. CIN1 (n = 64)
**Predictors/model**	AUC (95% CI)	AUC (95% CI)	AUC (95% CI)
***CDKN2A***	0.93 (0.90, 0.96)	0.97 (0.94, 0.99)	0.89 (0.85, 0.94)
***KRT7***	0.85 (0.80, 0.90)	0.89 (0.85, 0.94)	0.80 (0.74, 0.87)
***ARG1***	0.68 (0.61, 0.75)*	0.72 (0.65, 0.80)*	0.62 (0.54, 0.71)*
***HLA-DQB2***	0.57 (0.50, 0.64)*	0.69 (0.61, 0.76)*	0.54 (0.46, 0.63)
^a^ *CDKN2A* + *KRT7* + ARG1* + *HLA*-DQB2*	0.95 (0.93, 0.97) *p* = 0.027	0.99 (0.97, 1.0) *p* = 0.027	0.91 (0.87, 0.95) *p* = 0.11
^b^ Best model	(*CDKN2A* + ARG1*) 0.95 (0.92, 0.97) *p* = 0.030	(*CDKN2A* + ARG1*) 0.98 (0.97, 1.0) *p* = 0.030 (*CDKN2A* + HLA-DQB2*) 0.98 (0.97, 0.99) *p* = 0.041	(*CDKN2A* alone) (reported above)
**HOP/LOP**			0.72 (0.66, 0.79)
**HOP/LOP + *CDKN2A***			0.91 (0.86, 0.95) *p* < 0.001^c^
**HOP/LOP + *KRT7***			0.86 (0.81, 0.91) *p* < 0.001^c^
HOP/LOP + ARG1*			0.78 (0.70, 0.85) *p* = 0.014^c^
**HOP/LOP + *HLA*-*DQB2***			0.76 (0.69, 0.84) *p* = 0.10^c^
^d^ Best model including HOP/LOP			(HOP/LOP + *CDKN2A*) (reported above)

Results from receiver operating characteristic (ROC) curve analyses. Results reported as area under the receiver operating characteristic curve (AUC) with 95% confidence intervals (CIs) and *p*-values from the DeLong test.

qPCR, quantitative polymerase chain reaction; HPV, human papilloma virus; HOP-HPV, high oncogenic potential HPV (HPV16, 18, 31, 33, 45, 52, and 58); CIN1–3, cervical intraepithelial neoplasia grade 1–3.

* Lower expression gave higher odds of CIN2/3.

a AUC for predicted probabilities from a logistic regression model using a combination of all biomarkers (log-transformed) and *p*-values for the comparison of the combined model with the model with just *CDKN2A*.

b Model including the combination of (log-transformed) biomarkers that attained statistically significantly better discrimination (*p* < 0.05) than *CDKN2A* alone, with *p*-values for the comparison of the combined model with the model with just *CDKN2A*, and where inclusion of more biomarkers did not give further improvement.

c *p*-Value for comparison with model with only HOP/LOP.

d Inclusion of more biomarkers did not give statistically significant improvement.

#### 3.4.3 Expression levels related to HPV groups

*CDKN2A* and *KRT7* showed significantly higher relative expressions in the HOP-HPV group (n = 127) compared to the LOP-HPV group (n = 55) with medians of 1.89 vs. 0.29 (*p* < 0.001) and 1.88 vs. 1.13 (*p* = 0.032), respectively. A statistically significantly lower expression was found for *ARG1* (*p* = 0.012), but for *HLA-DQB2*, there were no statistically significant differences (*p* = 0.82).

#### 3.4.4 ROC analysis with combined biomarker expression levels and HPV groups

**Figure 4:**
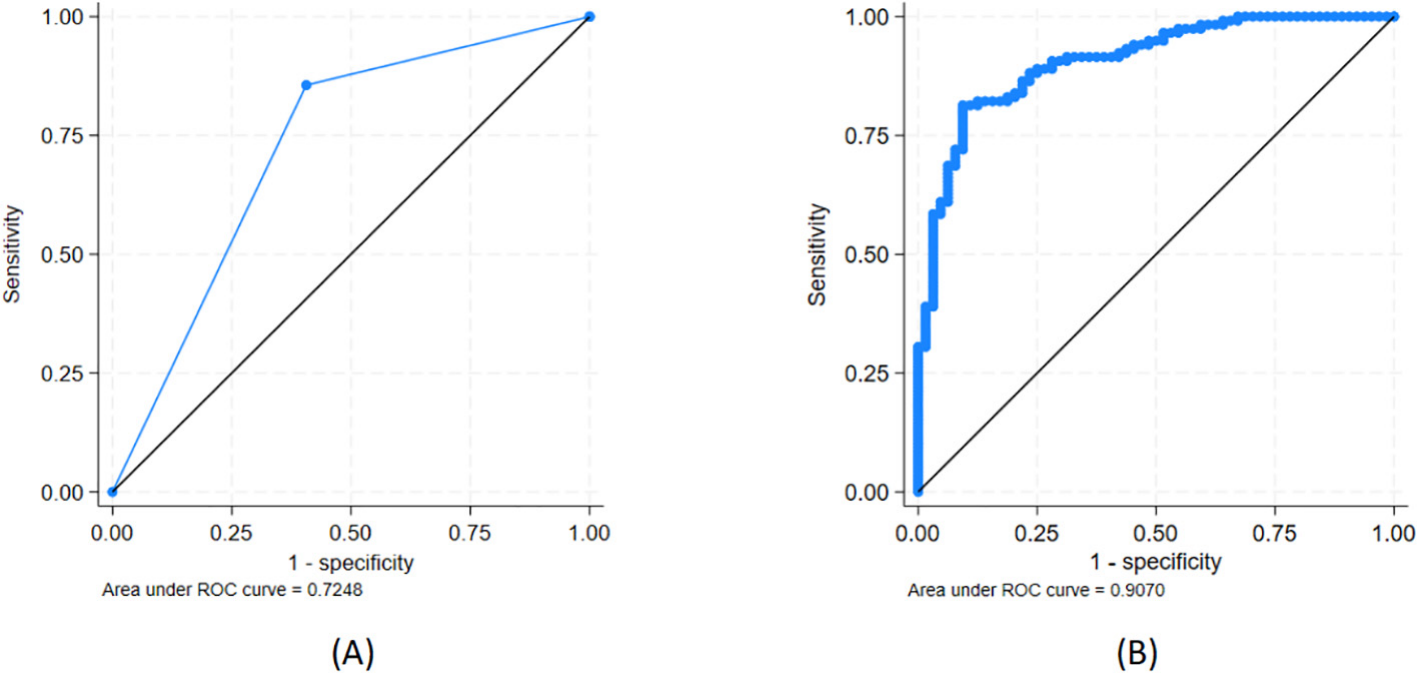
Ability to discriminate between CIN2/3 (n = 118) and CIN1 (n = 64) for **(A)** high/low oncogenic potential (HOP/LOP) of HPV genotype and **(B)** HOP/LOP + *CDKN2A*. See further details in **Table 3**.

HOP-HPV was associated with a positive predictive value for CIN2/3 (vs. CIN1) of 80%, whereas LOP-HPV was associated with a negative predictive value of 69%, and the ROC AUC was 0.72 (95% CI, 0.66–0.79) (see **Figure 4A**). The models using a combination of the HPV group (HOP/LOP) and (log-transformed) *CDKN2A*, *KRT7*, or *ARG1* were statistically significantly better at predicting CIN2/3 versus CIN1 compared to the HPV group alone (*p* < 0.001, *p* < 0.001, and *p* = 0.014, respectively), but not for *HLA-DQB2* (*p* = 0.10) (see **Table 3**).

The best model included the HPV group and log-transformed CDKN2A expression level and had an AUC of 0.91 (95% CI, 0.86–0.95) (**Figure 4B**). Adding either *ARG1* alone or together with *KRT7* to this model both led to improvements in the AUC, but not statistically significant improvements (*p* = 0.11 and *p* = 0.11, respectively).

## 4 Discussion

Many national screening programmes have implemented primary HPV testing as a substitute for primary cytology screening. The advantage is to detect women at risk in an early phase, but at the expense of overdiagnosis, with several unnecessary biopsies taken from HPV-positive women, diagnosed as CIN1 or normal and thus not subjected to immediate risk for progression to cancer (2). In a cohort of women following a CCSP, the main objective of the current study was to explore biomarkers to distinguish between women in need of immediate follow-up with biopsy and those who would benefit from a less invasive follow-up with cytology. Based on results from the Oncomine™ Immune Response Research Assay, the expressions of the immune markers *ARG1* and *HLA-DQB2* and the tumour markers *CDKN2A* and *KRT7* were evaluated via qPCR in a cohort of 264 biopsies with normal, CIN1, CIN2, or CIN3 biopsies in the follow-up of women with abnormal cytology and/or hrHPV infection at baseline cytology. To identify possible biomarkers, differences in gene expression, alone or in combination with information regarding the high or low oncogenic potential of the HPV types detected in the biopsy, were tested.

In an earlier NGS transcriptomic study (25), our group demonstrated differences between 14 normal and 13 CIN3 biopsies regarding the expression of genes related to a favourable or non-favourable immune response, tumour markers, and cell cycle checkpoints. The current extended NGS transcriptomic study analysed 28 CIN3 and 14 normal biopsies, and 22 differentially expressed genes were identified, all showing statistically significant *p*-values and FDR-adjusted *p*-values. However, apart from *HLA-DQB2*, genes with the highest fold-change in the transcriptomic study coincide with those found in the current study and confirm the high sensitivity of the Oncomine™ Immune Response Research Assay, even in small cohorts (30).

The expression of tumour markers *CDKN2A* and *KRT7* by qPCR was found to be significantly higher in biopsies positive for HOP-HPV as compared to the LOP-HPV group, while a significantly lower expression was found for the immune marker *ARG1*. Furthermore, to predict CIN2/3 versus CIN1, a combination of the HPV group (HOP/LOP) and the expression of *CDKN2A*, *ARG1*, or *KRT7* was statistically significantly better than the HPV group alone.

The current results confirm *CDKN2A* alone as the best marker to distinguish between CIN2/3 and CIN1. The tumour marker *CDKN2A*, with more than 23-fold higher expression in CIN3 versus normal biopsies, is a well-established immunohistochemical marker for grading of CIN1, CIN2, and CIN3 (32). Furthermore, *CDKN2A* was upgraded in HOP-HPV-positive biopsies. However, while the interpretation of immunohistochemical staining is subjective and time-consuming, measuring gene expression by use of NGS (33) or qPCR (31, 34) is much more precise, requires less hands-on time, and has become accessible in many pathology departments. *CDKN2A* inactivates the pRb with subsequent loss of cell cycle control, genome instability, and increased proliferation followed by transformation to cancer (35).

*CDKN2A* together with either *HLA-DQB2* or *ARG1* was the best model to discriminate CIN2/3 versus normal biopsies.

*HLA-DQB2* (paralogue to the *HLA-DQB1*) with 3.6-fold higher expression in normal versus CIN2/CIN3 is a class II human leukocyte antigen (HLA), explicitly expressed on Langerhans cells (LCs) (36, 37). LCs are the sentinel DCs and the most important antigen-presenting cells in the stratified squamous epithelium in the cervical mucosa and are crucial for the clearance of virus-infected cells. IFN-α, a type I interferon, is produced in large quantities by DCs and is essential for clearing virus infections and promoting recruitment of monocytes into the inflammatory site, where they differentiate into antigen-presenting cells (APCs) (38). Most sexually active women incur an HPV infection during their lifetime; however, more than 90% are transient infections cleared by innate immune responses (1, 39). The current results confirm the importance of defeating a chronic inflammation and thereby establishing a favourable first-line defence with high numbers of antigen-presenting LCs. In line with our results, a recent study on cervical cancer and recurrence elucidated the context between high expression of *HLA-DQB1* and high survival rate, as compared to patients with low expression of *HLA-DQB1* (40).

CIN2/3 is defined as pre-cancer, and the current transcriptomic study demonstrates increased expression of genes related to pro-tumorigenic immune responses in the microenvironment of these biopsies (**Table 2**). Interestingly, *CCL20* is involved in the migration of Th17 cells into tumour tissue in cervical cancer and contributes to immunopathogenesis (41, 42). *TNFRSF18* encodes the glucocorticoid-induced tumour necrosis factor receptor-related protein (GITR) with the potential to differentiate and expand the population of T-regulatory lymphocytes (Tregs) (43–45). High expression of the B cell-specific co-activator *POU2AF1* (46) and high expression of the chemokine *CXCL13*, associated with tumour metastasis, are both involved in the upregulation of the B-cell population (47). Altogether, the results from the transcriptomic study show clear signs of a Th2-driven inflammation, associated with the development of a carcinogenic microenvironment (CEM) (45, 48, 49).

The development of chronic inflammation is one of the molecular mechanisms involved in the evasion of anti-tumour immunity. In tumours, the most abundant myeloid cells activate Th2 cytokines, IL-10 and TGF-β, stimulate the arginase pathway, and suppress nitric oxide synthase (iNOS) expression. Thereby, differentiation of the so-called tumour-associated macrophages is induced, associated with high expression of *ARG1* (50).

During chronic inflammation, the endogenous origin of the amino acid l-arginine (L-arg) is strongly depleted. Lack of L-arg during cancer development has been linked to dysfunctional immune responses with inhibition of a favourable T-cell response and accumulation of myeloid-derived suppressor cells (MDSCs) and thereby functional impairment of T cells and poor prognosis (51, 52). In various cancer types, increased expression of presumably dysfunctional *ARG1* was associated with poor overall survival rates, development of suppressive M2-like phenotype, and an immunosuppressive microenvironment (50).

In contrast, *ARG1* with the highest expression fold-change (34-fold) in normal versus CIN3 and downregulated in biopsies positive for HOP-HPV has also been identified as a suppressive mediator of a Th2-dependent inflammation (53). In our study, the expression of *ARG1* was entirely absent in most CIN2/CIN3 biopsies and therefore incapable of eliminating a Th2-response as observed in high-grade CIN (45, 48). A former study by our group identified higher expression of *ARG1* in normal as compared to CIN3 biopsies (25), but to our knowledge, the current study is the first to demonstrate an association between the expression of *ARG1* and high/low oncogenic potential HPV.

In normal biopsies, four times higher expression was found for the chemokine receptor *CX3CR1*, which was found to play a pivotal role together with *CX3CL1* (fractalkine) during the activation of NK cells (54, 55). Fractalkine is expressed by immune effector monocytes and promotes their ability to mediate migration from blood to tissue where they differentiate into inflammatory DCs and macrophages and promote the activation of NK cells (56). Normal biopsies also had nearly ninefold higher expression of *NCAM1*, a marker for NK cells, which belong to the innate immune response system, and, together with DCs, are crucial as first-line defence against infections. Together with high expression of *ARG1*, these are all signs of a favourable Th1 immune response, and by generating IFN-γ and the transcription factor IRF1, Th1 positively regulates iNOS, responsible for converting l-arginine into nitric oxide (NO). In addition to increasing the tumour suppressor p53, NO is an important mechanism for regulating T cells during the early stage of immune responses against DNA and RNA virus infections. Relative to its concentration, NO triggers different effector mechanisms, and low concentrations stimulate a Th1 response. Furthermore, NO is also capable of regulating humoral responses by inhibiting the expression of the B-cell activating factor (57). On the contrary, compared to HPV-negative controls, high concentrations of iNOS, and subsequently also NO, have been found to be positively correlated with tumorigenesis and lymph node metastasis in cervical cancer patients and hrHPV-positive women (58).

In order to complete their life cycle, hrHPVs have evolved mechanisms to evade host immune signalling by epigenetically interfering with the methylation status of genes involved in the immune response, thereby causing immune evasion and suppressed antitumour immune responses (15–17, 19, 20, 22). Combined with high expression of *CDKN2A* and subsequent upregulation of the host cell cycle and proliferation-related genes in HOP-HPV-positive biopsies, this could be a hallmark for the transformation of a transient to persistent HPV infection and an important step towards the development of cancer.

*KRT7*, showing higher expression in CIN3 versus normal biopsies, encodes Keratin7 (KRT7) and is identified as a marker for squamocolumnar junction (SCJ) cells, located at the interface of the transformation zone (TZ) in the cervix (59). KRT7 belongs to a group known as “simple epithelial keratins” found in tissue with single-layered stratified epithelia (60). The SCJ cells were described to have a unique gene expression profile and morphology with embryonic characteristics, distinct from the stratified squamous epithelium in the TZ. In line with our findings, strong immunostaining for KRT7 has been found in 90% of high-grade squamous intraepithelial lesions (HSIL or CIN2/3) and cervical carcinomas (61). Emerging evidence confirms that SCJ cells are involved in an early stage of HPV-related CIN2/3 development and cervical carcinogenesis (62).

Despite that HPV over the last decades has become ubiquitous, dependent on HPV genotype, women’s age, and viral load, most infections with histopathology proven normal or CIN1 biopsies are cleared by the immune system (63, 64). CIN1 is infected with free episomal HPV expressing E4, whereas E4 expression is lost in CIN3. In fact, E4 has been suggested as a biomarker to distinguish between CIN2 and CIN1 (65). As the HPV E4 protein interacts with filaggrin, a cytokine-binding protein, the loss of cytokeratin will eventually incur a collapse of the matrix. Microscopically, CIN1 is characterized by squamous epithelial cells known as koilocytes harbouring enlarged hyperchromatic nuclei surrounded by a perinuclear halo observed in differentiated epithelial layers (66). Despite confirmation by an expert pathologist of CIN1 in the adjacent sections after DNA isolation, 36% of CIN1 were HPV negative. The high percentage of HPV-negative CIN1 is most likely due to a low virus copy number (10), below the detection level for the Anyplex Genotype assay. Thirteen (16%) of the normal biopsy cohort were genotyped by the INNO-LiPA assay: 10 (77%) were HOP-HPV positive, while three (23%) were LOP-HPV positive. INNO-LiPA is a very sensitive method and is capable of detecting HPV in very low copy numbers (26). The majority of women included in the study had either high-grade cytology (HSIL/ASC-H) or a proven HPV-positive cytology, with a median of 41 days, prior to the biopsy. Furthermore, HPV detected in biopsies diagnosed as normal and as so show no signs of an HPV infection are most likely HPV in a latent state (67, 68).

A recent systematic review and meta-analysis found regression in 60%, 55%, and 28% of CIN1, CIN2, and CIN3, respectively, and progression in 11% and 19% of CIN1 and CIN2, respectively (69). A study with long follow-up of women who were deprived of the opportunity to be treated revealed that approximately 30% of high-grade CIN3 developed into cancer (70). Based on the incidence rate for CIN1 or normal conization specimens, a retrospective study calculated more than a 27% regression rate for CIN2/3 within 2 years for hrHPV-positive women (71). Regarding follow-up of women attending CCSP, the fact that a low number of CINs progress to cancer is troublesome for decision-making. Undoubtedly, many women harbouring transient HPV infections with low oncogenic potential (7, 8) are exposed to overdiagnosis and overtreatment, so more precise biomarkers to distinguish between HPV infections with high or low oncogenic potential are required.

This study illustrates that follow-up with a biopsy limited to women infected with HOP-HPV and detected with biomarkers showing impaired immune response and increased proliferation of HPV-infected cells is more beneficial as compared to the current partial genotyping of HPV16 and 18 and “12 other” HPV as a group. The promising markers *CDKN2A*, *KRT7*, *ARG1*, and *HLA-DQB2* were detected in biopsies, but to be useful as markers for women in need of follow-up with potential biopsy, they need to be validated in liquid-based cytology specimens. Del Pino et al. explored the mRNA expression of *CDKN2A*, *BIRC5*, *MMP9*, *TOP2A*, *MCM5*, and *MKI67* in RNA isolated from ThinPrep cytology specimens for the detection of women harbouring HSIL lesions. The results were promising, and the approach proved useful (72).

Despite retesting, one CIN3 case, with CIN2 in the adjacent section after DNA isolation and CIN2 in the follow-up cone, was HPV negative; however, as characterized by high expression of *CDKN2A* and *KRT7*, low expression of *HLA-DQB2*, and zero *ARG1* expression, this was clearly a CIN2/CIN3 case.

A limiting factor for qPCR is the decision of how many genes to validate. Additionally, the amount of high-quality RNA isolated from CIN2/3 and CIN1 lesions was limited. *NCAM1* (neural cell adhesion molecule 1), also known as *CD56* and a marker for NK cells, was a relevant gene to validate with almost ninefold higher expression in normal biopsies in the transcriptomic study. However, after analysis of 50 normal and 68 CIN3 biopsies by qPCR, no statistically significant differences were found between the groups. The downregulated *NCAM1* observed in CIN3 biopsies in the transcriptomic study is expected, as phenotypic alterations and functional deficiencies of NK cells have been found in patients harbouring HPV16 infections and cervical cancer (73). However, inhibition of NK cell cytotoxicity has been proven to be related to an immunosuppressive milieu, as observed in the CIN3 biopsies (74). Regulatory NK cells (NKregs) were described to have high expression of *CD56* and express *IL-10*, but not *IFN-γ*, and characterize a subset of NK cells, which induce chronic inflammation with immunosuppressive properties (75, 76). Cytotoxic NK cells have low expression of *CD56* and express *CD16*, *CXCR1*, and *CX3CR1*, with the latter associated with a favourable immune response and with more than fourfold higher expression in normal biopsies. One possible explanation for the failure to find differences between groups in the current qPCR study could be when testing a larger sample cohort, as the effect of NKregs with high expression of *CD56* in CIN3 camouflages the higher number of NK cells with lower *CD56* expression in normal biopsies.

*CX3CR1* was also a relevant gene for validation; however, a better FDR-adjusted *p-*value and *p*-value supported the decision to validate *HLA-DQB2*. The two genes are closely connected, as *HLA-DQB2* is expressed on DCs (36), while *CX3CR1* promotes the differentiation of DCs and the activation of NK cells (55).

The number of biopsies used for validation of the transcriptomic study, especially in the CIN1 group (N = 64), is a limitation of the study. Due to issues through fixation, embedding, and storage processes, variable qualities of archival FFPE tissue will have an impact on the quality and quantity of isolated RNA (77). The current study is based on targeting quantification of the expression levels of genes and requires the exact amplifiable RNA concentrations from all FFPE samples used in the study. To ensure non-variation between the patient cohorts or the different diagnoses, the amplifiable concentration was measured using a functional RNA quantification assay. However, unfortunately, this RNA quality and quantitative testing also resulted in many samples containing less than the required RNA concentration (10 ng/µL) and therefore had to be discarded. Consequently, this had an impact on the number of biopsies suited for the study.

In conclusion, the current study demonstrates how low expression of the immune marker *ARG1* and high expression of the tumour markers *CDKN2A* and *KRT7* correlate with the group of high oncogenic potential HPV and the development of high-grade CIN. Furthermore, a model using expressions of *CDKN2A*, *KRT7*, or *ARG1* combined with HPV grouping for predicting CIN2/CIN3 versus CIN1 was significantly better as compared to HPV grouping alone, and *CDKN2A* together with either *HLA-DQB2* or *ARG1* was the best model for predicting CIN2/3 versus normal. These results demonstrate automated targeted specific probe-based qPCR assays as reliable for quantification of gene expression and suitable for measuring gene expression in a limited number of genes. qPCR is less expensive and more flexible as compared to assays performed on fully automated NGS devices.

The unnecessary use of diagnostic biopsies for women attending CCSP implies overworked staff in pathology departments. Additionally, the anxiety among many women linked to taking a biopsy should not be underestimated. Taking this into account, follow-up with a non-invasive cytology test of women not diagnosed with HSIL cytology nor high oncogenic potential HPV positivity would be beneficial. For triage of this group of women, the use of mRNA expression of *CDKN2A*, *KRT7*, *HLA-DQB2*, or *ARG1*, combined with specific HPV genotyping of the high or low oncogenic potential HPV groups, could increase the safety regarding decision-making for correct interventions.

## Data Availability

In line with rules made by the Norwegian Regional Ethics Committee, data availability of the gene expression data generated in this study are not made publicly available in a repository due to privacy restrictions, but will be made available upon reasonable request to the corresponding author irene.tveiteras.ovestad@sus.no.
